# An Adaptive Super-Resolution Network for Drone Ship Images

**DOI:** 10.3390/e28020187

**Published:** 2026-02-07

**Authors:** Haoran Li, Wei Xiong, Yaqi Cui, Libo Yao

**Affiliations:** Naval Aviation University, Yantai 264001, China; rizhaolihaoran@163.com (H.L.); cui_yaqi@126.com (Y.C.); ylb_rs@126.com (L.Y.)

**Keywords:** image super-resolution, adaptive learning, drone ship images, information theory

## Abstract

Uncovering latent structures from complex, degraded data is a central challenge in modern unsupervised learning, with critical implications for downstream tasks. This principle is exemplified in the domain of aerial imagery, where the quality of images captured by drones is often compromised by complex, flight-induced degradations, thereby raising the information entropy and obscuring essential semantic patterns. Conventional super-resolution methods, trained on generic data, fail to restore these unique artifacts, thereby limiting their effectiveness for vessel identification, a task that fundamentally relies on clear pattern recognition. To bridge this gap, we introduce a novel adaptive super-resolution framework for ship images captured by drones. The approach integrates a static stage for foundational feature extraction and a dynamic stage for adaptive scene reconstruction, enabling robust performance in complex aerial environments. Furthermore, to ensure the super-resolution model’s generalizability and effectiveness, we optimize the design of degradation methods based on the characteristics of drone aerial images and construct a high-resolution dataset of ship images captured by drones. Extensive experiments demonstrate that our method surpasses existing state-of-the-art algorithms, confirming the efficacy of our proposed model and dataset.

## 1. Introduction

With the rapid advancement of drone technology, drones have been widely adopted in various fields, such as agricultural production [1], natural disaster monitoring [2], and military reconnaissance [3], owing to their flexibility and timeliness in information acquisition. However, the unique operational profile of drones introduces a constellation of severe and often intertwined degradation challenges that are far more complex than those encountered in conventional ground-based photography. Limitations in onboard sensor size and payload capacity often necessitate the use of less-than-optimal optics, directly compromising image clarity. Furthermore, the inherent instability of the aerial platform, even with advanced gimbals, induces unavoidable motion blur, which is exacerbated during high-speed maneuvers or in turbulent atmospheric conditions. Weather phenomena such as haze, fog, and atmospheric turbulence are not mere obstacles but active participants in the degradation process, scattering light and reducing contrast over long distances. These compounded factors result in acquired images exhibiting low resolution, blurred details, and significant noise, thereby fundamentally obscuring the underlying semantic structures and latent patterns within the data, posing a significant barrier to downstream tasks that rely on robust feature extraction and clustering [4,5].

Single-image super-resolution (SR) is able to enhance the visual quality of an image by applying image restoration techniques to extrapolate and reconstruct high-frequency details from the limited information inherent in a low-resolution (LR) source [6]. Super-resolution has emerged as a prominent area of investigation, with deep learning catalyzing a paradigm shift and achieving unprecedented performance gains. The ability of deep neural networks to implicitly learn prior distributions has significantly enhanced SR performance, and there has been a dramatic upsurge in the use of deep networks to achieve effective super-resolution [7,8,9,10]. Moreover, recent studies have successfully applied SR technology to various fields, including remote sensing [11], medical science [12] and face recognition [13]. Nevertheless, the majority of these state-of-the-art deep learning-based SR methods suffer from a critical domain gap when applied to real-world drone imagery. This limitation stems primarily from their training paradigm: most models are exclusively trained and evaluated on synthetic data generated from a single, idealized degradation model—typically, the simple bicubic down-sampling kernel [14]. This controlled environment creates a significant bias, as the models learn to reverse a specific, known degradation pattern rather than the complex and unknown combinations prevalent in aerial scenarios. Consequently, when confronted with real drone images, these models exhibit a stark performance drop, which severely hinders their practical applicability.

Image super-resolution techniques can allow the drone to fly higher and cover larger areas while still maintaining high image quality. As far as drone images are concerned, the perspective is often from above, which differs from the human perspective of natural images. In addition, drones face more complex and diverse imaging scenes, such as long-distance photography, high-magnification zoom, extensive maneuvering, and varying weather conditions, all of which contribute to their distinctive features and increase information entropy, which may lead to differences between drone-image super-resolution research and conventional methods.

Nevertheless, research on super-resolution reconstruction for drone aerial images is still in its infancy, lacking support from relevant publicly available datasets. In particular, the data for ship scenes captured by drones is even more scarce; as ships are the main carriers operating on the water surface and a key focus of maritime reconnaissance, conducting research using drone aerial ship images holds significant practical importance.

Based on the above considerations, a novel adaptive super-resolution method for drone-captured ship images is proposed in this paper. It is mainly composed of a simple degradation parameter estimation module and an adaptive reconstruction module. Furthermore, we construct a drone-captured ship image super-resolution dataset and optimize the design of degradation methods according to the characteristics of drones. Our main contributions can be summarized as follows:

(1) An adaptive super-resolution method for drone-captured images is introduced, which adopts a strategy composed of static and dynamic parts to handle the super-resolution reconstruction problem in complex scenes.
(2) A degradation process is designed to model practical degradations in drone aerial images, considering realistic conditions during drone capture.
(3) A novel dataset of ship images captured by drones is introduced, which contains high-resolution drone images. Through extensive experiments and comparisons, our method achieves better performance than previous works.

The rest of this paper is organized as follows. Section 2 presents the related work. Section 3 details the methodology. Section 4 introduces experimental details. Section 5 reports the results of our experiments. Finally, Section 6 concludes our method and suggests directions for future research.

## 2. Related Work

### 2.1. Drone Image Super-Resolution

Owing to the extensive integration of drones into various industries, methods in the field of computer vision have emerged as a fundamental enabler of their functionality [15,16,17]. By enhancing image clarity, drone super-resolution facilitates high-precision downstream tasks, including surveillance and reconnaissance [18]. Driven by the superior performance of deep learning, current research in image super-resolution has largely moved beyond traditional interpolation and reconstruction methods [19], which are now seldom employed due to their inherent performance limitations. The predominant paradigm in deep learning-based image reconstruction relies on Convolutional Neural Networks (CNNs) or Generative Adversarial Networks (GANs). However, the majority of studies have concentrated on natural images [20], which are typically captured from a horizontal, human-like perspective. In contrast, aerial drone images are typically captured from a top-down or oblique angle, which gives them a distinctive perspective compared to conventional natural images. Currently, research on super-resolution for drone images remains limited. Lin et al. [21] constructed a super-resolution dataset by capturing drone images at various altitudes, and a height-aware framework was proposed. Zhao et al. [22] introduced a multi-conditioned guidance network for the super-resolution of thermal drone images utilizing detailed information from visible images. To support this work, the authors also curated a paired dataset of drone visible and thermal imagery. Zhao et al. [23] further expanded the diversity and scale of the aforementioned dataset and subsequently proposed a guided decoupling network for the super-resolution task. Han et al. [24] proposed a cross-platform super-resolution reconstruction method for remote sensing images, which leveraged high-resolution (HR) drone aerial remote sensing images to guide the reconstruction of low-resolution satellite images. Li et al. [25] introduced an efficient image restoration algorithm based on diffusion models, which utilizes the frozen internal representations of pretrained encoder–decoder networks to restore high-resolution aerial images. Weng et al. [26] proposed a Gaussian quantization representation learning method oriented to diffusion models for few-shot drone-captured infrared image super-resolution. However, research in this domain is still in its infancy, and current solutions lack the adaptability required for diverse and challenging aerial scenarios. To overcome these limitations, we introduce a robust and flexible framework for the reconstruction of drone images, contributing to the practical applicability of super-resolution in real-world drone images.

### 2.2. Image Degradation Models

As mentioned in the Introduction, most SR methods [20,27,28] employ bicubic down-sampling or other simple degradation models for training and evaluation. The complexity of real-world degradation, however, poses a significant challenge to models trained on simplistic assumptions, limiting their effectiveness on real-world images. These approaches where the degradation model is known or assumed is termed non-blind super-resolution. In contrast, blind super-resolution addresses the more challenging scenario where the degradation process is unknown, making it particularly suitable for real-world images [29]. To that end, a few methods have been proposed. Gu et al. [30] introduced an iterative kernel correction approach, which progressively refines the degradation kernel within the super-resolution process to improve reconstruction quality. Zhang et al. [31] designed a random shuffle strategy to synthesize more practical degradations, including blur, noise, down-sampling, and JPEG compression. Wang et al. [32] proposed a high-order degradation modeling process and incorporated sinc filters in the synthesis process. Wang et al. [33] proposed an unsupervised degradation representation learning method. Through a dedicated degradation perception module, it fuses these degradation features to enhance the model’s reconstruction performance under unknown degradation conditions. Liang et al. [34] introduced an efficient degradation-adaptive network, which improved the adaptability to diverse degradations through the joint optimization of multiple experts. In essence, while blind super-resolution has made strides, most approaches rely on generic degradation models. However, drones routinely face challenging scenarios, from high-speed maneuvers to adverse weather, which imprint a complex signature of degradation on the captured images. This gap between generic modeling and the distinct realities of drone imagery severely compromises performance. The degradation model proposed in our work is a step in this direction.

## 3. Methodology

**Figure 1:**
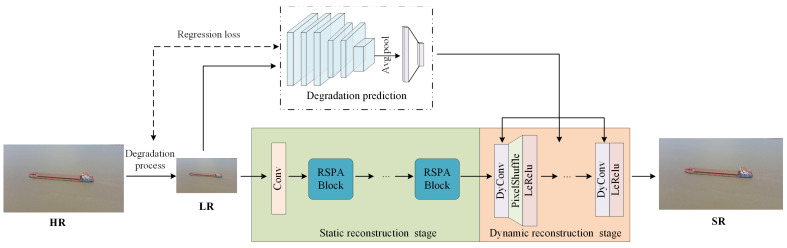
The overall framework of the proposed network.

As shown in Figure 1, the adaptive super-resolution network for drone-captured ship images proposed in this paper comprises two core modules: a degradation prediction module and an adaptive reconstruction module. Initially, the degradation prediction module analyzes the input low-resolution image to estimate its specific degradation pattern. This degradation information is then fed into the adaptive reconstruction module, which employs a hybrid “static + dynamic” strategy. The process begins with the static feature reconstruction stage. This stage functions as a general-purpose super-resolution network, processing the input LR image to extract fundamental textural details and structural information. Its primary role is to generate a preliminary high-resolution feature map that captures the essential content of the image, independent of the specific degradation type. Crucially, this static feature map then serves as the foundation for the dynamic stage. The dynamic stage takes both the static features and the degradation parameters predicted by the degradation prediction module as inputs. It leverages the degradation information to selectively modulate and enhance the static features. For instance, if the predictor indicates severe motion blur in a specific direction, the dynamic stage will apply a learned operation to counteract the degradation. In essence, the dynamic stage acts as an intelligent “adapter” that tailors the generic restoration from the static stage to one that is adapted to the degradation state of the input image. By fusing these two complementary stages of information, our model can robustly and flexibly restore a clear, high-resolution image from a wide variety of real-world drone degradations.

### 3.1. Degradation Model

**Figure 2:**
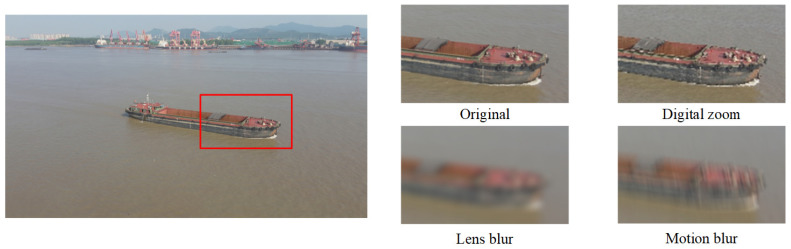
Schematic illustration of different imaging conditions.

While significant efforts have been dedicated to the development of degradation models for real-world scenes, existing approaches often employ a generic series of processing steps, such as noise injection, blurring, and down-sampling, without adequately accounting for specific circumstances. To more accurately reflect the actual flight status of drones, we have enhanced existing degradation models [34] by incorporating factors specific to aerial photography, such as motion blur, lens blur, and digital zoom. Therefore, the model can more accurately simulate the complex conditions of real-world data. Figure 2 shows some samples in these cases.

Currently, most image degradation models employ Gaussian blur, which is characterized by its uniform distribution, to simulate blurring effects. This approach is suitable for modeling the lens blur caused by defocus during drone flight. Gaussian blur is defined as follows:

(1) $$g\left(x,y\right)=\frac{1}{2\pi \sigma }\exp\left(-\frac{x^{2}+y^{2}}{2\sigma^{2}}\right)$$

where $g\left(x,y\right)$ is the weight of the Gaussian blur and σ is the standard deviation. To reflect the variability of real-world flight conditions, we do not use a fixed σ. Instead, for each training image, $\sigma ∼U\left(\sigma \_\min,\sigma \_\max\right)$ is randomly sampled from a uniform distribution. This range is chosen to simulate blur ranging from slight defocus to more significant lens softness.

However, the captured images are also susceptible to motion blur induced by either target motion or drone maneuvering. Unlike uniform Gaussian blur, motion blur is directional in nature, and its function is modeled as follows:

(2) mx,y=1L,x=−ytanθx2+y2≤2L0,others

where $m\left(x,y\right)$ is the weight of the motion blur, L represents the distance an object moves (kernel size) and θ represents the angle of movement. Similarly, to model the unpredictability of drone dynamics and target movement, the motion blur parameters are randomized. The kernel length is sampled from $U\left(7,21\right)$ pixels to represent motion from slight jitter to significant displacement, and the angle (θ) is uniformly sampled from $U\left(-\pi ,\pi \right)$ to account for all possible directions of drone travel or maneuvering.

**Figure 3:**
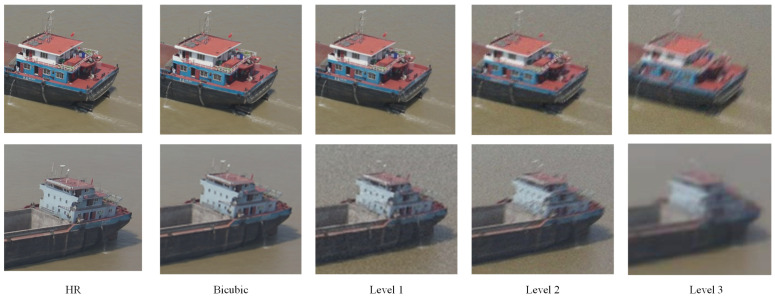
Schematic illustration of levels of degradation.

Drawing inspiration from prior works [31,34], we design three different levels of a degradation pipeline to simulate complex and realistic image degradation. While the foundational concept of a multi-stage approach draws from prior work, such as [34], our implementation is specifically tailored to address the unique challenges presented in this paper. A key distinction lies in our redefined parameter spaces; for instance, rather than employing generic blur kernels, we specifically model the mentioned motion and lens blur to accurately represent the physical degradations inherent to drone imagery, such as platform dynamics and optical aberrations. We also significantly expanded the standard deviation range for these blur kernels, ensuring the model is robust to both subtle and severe blurring artifacts. We also introduce a novel step to simulate digital zoom when degradation modeling; this is achieved by applying high-ratio down-sampling, followed by up-sampling, which effectively mimics the loss of detail inherent in digital magnification. For the input image (I) and scale factor (s), the zoom processing can be expressed as $I_{z}\;=\mathrm{Upsample}\left(\mathrm{Downsample}\left(I,s\right),1/s\right)$. Recognizing that imagery is rarely artificially up-sampled, we constrain the resizing process to two realistic scenarios: down-sampling or maintaining the original resolution. This structured pipeline sequentially applies these tailored degradations—blurring, resizing, noise injection, and aggressive JPEG compression—to generate highly realistic training data. These tailored modifications detailed in Table 1 ensure our synthetic data more closely mirrors the real-world distribution, thereby enhancing the model’s robustness and effectively bridging the domain gap between synthetic training data and real-world drone imagery. With respect to the specific parameter scale of the three levels presented in the table, level 3 includes two processing stages, and $\omega_{c}$ is the cutoff frequency of the sinc kernel. During training, we randomly sample from these methods according to a balanced probability distribution of [0.3, 0.4, 0.3] to generate the LR–HR image pairs. The blur operation employs motion and lens blur in equal proportions. Figure 3 shows samples with different levels of degradations.

The degradation prediction module is built upon a simple convolutional neural network architecture. Within this module, the intermediate layer of the linear mapping is set to a dimension of 64. To effectively guide the model in learning the degradation information from the input image, the network is trained with a regression loss. This loss function is given by

(3) $$L_{\mathrm{reg}}={\left\|\hat{y}-y\right\|}_{1}$$

where $\hat{y}$ represents the predicted degradation parameters and y represents the ground truth.

**Figure 4:**
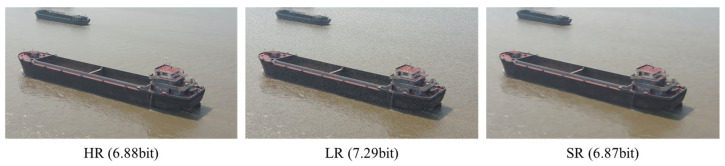
The Shannon entropy of a representative sample.

The degradations caused by drone flight may elevate the information entropy of an image, which, in turn, obscures its underlying semantic patterns. This phenomenon occurs because these degradations introduce random or complex artifacts, rendering the image content less predictable and more disordered. To quantify this effect, Shannon Entropy is employed. In Figure 4, we present the Shannon entropy values for the HR, LR, and SR images of a representative sample. The results demonstrate that image degradation can lead to an increase in entropy, whereas the entropy is markedly reduced after super-resolution reconstruction. This degradation-induced entropy increase is a general trend. However, we note an extreme case where severe blurring can paradoxically reduce entropy by averaging pixel values, though such instances are infrequent. The Shannon entropy of an image (I) can be defined as follows:

(4) $$H\left(I\right)=-\sum_{i=0}^{L-1}p\left(i\right){\;\log}_{2}p\left(i\right)$$

where L denotes the total number of possible gray levels in the image, i is the index of a specific gray level and $p\left(i\right)$ represents the probability of a pixel having gray level i.

### 3.2. Adaptive Reconstruction Module

The adaptive reconstruction module comprises two stages: static and dynamic feature reconstruction. In the static stage, the module focuses on extracting the textural details of the target from the input LR image, which provides a stable, content-rich foundation. Subsequently, based on these features, the dynamic stage leverages the predicted degradation information to guide the restoration process, ultimately producing a clear, high-resolution image. In this way, the two-stage approach adaptively achieves super-resolution for drone imagery.

**Figure 5:**
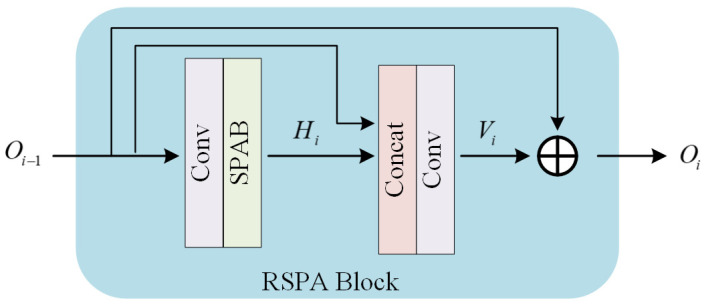
Architecture of the RSPA Block.

For the static reconstruction stage, we adopt swift parameter-free attention [35] which has demonstrated exceptional efficiency in super-resolution tasks by effectively enhancing salient features and suppressing redundant information. To make the reconstruction process robust, we design a Residual Swift Parameter-free Attention Block (RSPA Block) in this stage. As shown in Figure 5, RSPA Block incorporates swift parameter-free attention into the standard residual block, thereby enhancing the robustness of feature learning. Concatenating multiple blocks enables the comprehensive extraction of low-level features from the input image while simultaneously capturing both local and global characteristics. Then, the RSPA block can be expressed as follows:

(5) $$\begin{array}{l} O_{i}=O_{i-1}⊕V_{i}\\ V_{i}=\varphi \left(\mathrm{Concat}\left(H_{i},O_{i-1}\right)\right)\\ H_{i}=\psi \left(\varphi \left(O_{i-1}\right)\right) \end{array}$$

where ⊕ represents the element-wise sum, φ stands for the convolutional layer with a 3 × 3 kernel, ψ is the swift parameter-free attention block and $\mathrm{Concat}\left(•\right)$ represents the cat operation between feature maps in the channel dimension.

In the dynamic reconstruction stage, dynamic convolution [36], which can dynamically adjust parameters according to different inputs, is introduced. The core of our dynamic stage lies in its mechanism for generating convolutional weights, a process directly driven by the predicted degradation information. Specifically, the predicted degradation parameters ($\hat{y}$) are first fed into a fully connected network. This network acts as a learnable function (f) that maps the abstract degradation vector into a set of attention weights. These weights ($W=f\left(\hat{y}\right)$) determine the importance of different pre-defined convolution kernels in the subsequent step, and the number of convolution kernels in the bank is 5. The parameters of the dynamic convolution layer ($W_{\mathrm{dy}}=W*K$) are formed by a weighted combination of these kernels using the attention weights (W) and the weighting vector (K), which is adaptively modulated for the specific degradation of the input. The stage is introduced in the latter part of our model, enabling the model to combine the static features with the degradation information to achieve better reconstruction of low-resolution images.

This enables the network to adapt its behavior, significantly enhancing its capacity to address diverse and complex degradation scenarios. Consequently, our model can robustly generate high-quality SR images by adaptively tuning its internal parameters according to various input images. The process of dynamic convolution can be expressed as follows:

(6) $$F_{o}=W_{\mathrm{dy}}*F_{i}$$

where $W_{\mathrm{dy}}$ represents the parameters of dynamic convolution layer, $F_{o}$ and $F_{i}$ denote the input and output features and ∗ represents the convolution operation.

Furthermore, the dynamic reconstruction stage can be expressed as follows:

(7) $$\begin{array}{l} O^{\prime }=\gamma \left(\mathrm{PixelShuffle}\left(\xi \left(O\right)\right)\right)\\ I=\gamma \left(\mathrm{PixelShuffle}\left(\xi \left(O^{\prime }\right)\right)\right) \end{array}$$

where γ denotes the LeakyReLU activation function and ξ denotes the process of dynamic convolution.

Finally, the obtained I is processed by a dynamic convolution operation to map to the output dimension; then, $I_{SR}$ is generated by fusing the bicubic up-sampled input images with the output.

### 3.3. Loss Function

Commonly used loss functions reported in previous SR works [31,32,37] include pixel loss ($L_{\mathrm{pix}}$), adversarial loss ($L_{\mathrm{adv}}$), and perceptual loss ($L_{\mathrm{per}}$).

Pixel loss aims to align the spatial geometry of the reconstructed image with its original HR counterpart as far as possible. It is defined as follows:

(8) $$L_{\mathrm{pix}}={\left\|I_{SR}-I_{HR}\right\|}_{1}$$

where $I_{SR}$ denotes the SR image and $I_{HR}$ denotes the HR image.

Adversarial loss guides the network to reconstruct visually favorable images, and the U-Net discriminator with spectral normalization is adopted. The adversarial loss is defined as

(9) $$L_{\mathrm{adv}}=-\log\left(D\left(G\left(I_{LR}\right)\right)\right)$$

where $I_{LR}$ denotes the LR image, D denotes the discriminator and G denotes the generator.

Perceptual loss enhances the perceptual quality of SR images by minimizing the feature distance between SR and HR images in a deep feature space, and a pretrained VGG19 network [38] is used to extract the features. It is defined as

(10) $$L_{\mathrm{pre}}={\left\|\phi_{i}\left(I_{SR}\right)-\phi_{i}\left(I_{HR}\right)\right\|}_{1}$$

where $\phi_{i}$ denotes the *i*th layer output of VGG19.

In addition to the above loss functions, we adopt the regression loss mentioned in Section 3.1, which is computed for the degradation prediction. The total loss is defined as follows:

(11) $$L=L_{\mathrm{pix}}+L_{\mathrm{adv}}+L_{\mathrm{pre}}+L_{\mathrm{reg}}$$

## 4. Experimental Details

### 4.1. Experimental Settings

**Figure 6:**
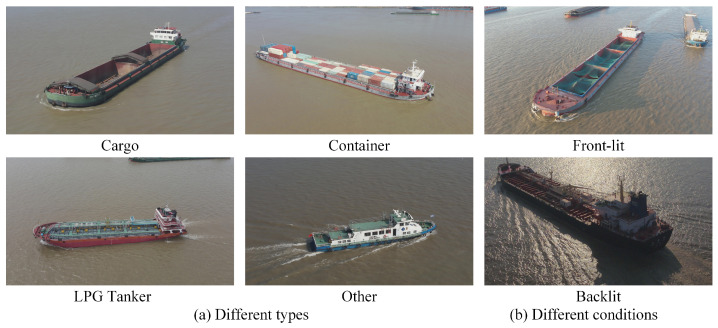
Some image samples from our dataset.

Given the paucity of publicly accessible aerial drone imagery of ships, we construct a drone aerial ship image dataset by conducting real data collection experiments. This dataset comprises high-resolution imagery captured by a DJI M30T drone (DJI, Shenzhen, China), encompassing a diverse array of ship classes; some image samples are displayed in Figure 6. For this study, the training partition consists of 7260 images, and the test partition contains 106 images. Specifically, the dataset encompasses diverse vessel types, including cargo ships, container ships, LPG tanker ships, and other marine vessels. It comprises images captured under varied scenarios, such as sunny weather, cloudy weather, front-lit conditions, and backlit conditions, thereby ensuring the data exhibits substantial diversity. During the model training process, the training HR patch size is set to 256 × 256, the learning rate is set to $1\;\;\times \;e^{-4}$ and the total batch size is 8. We adopt the Adam optimizer [39] with $\beta_{1}=0.9$ and $\beta_{2}=0.99$ to optimize the model. We first train our model for 500 K iterations only with the pixel loss. Then, we use all the training losses to train the obtained model for 200 K iterations.

### 4.2. Evaluation Metrics

To evaluate the effectiveness of the super-resolution task quantitatively, we employ the peak signal-to-noise ratio (PSNR) [40,41], the structural similarity index metric (SSIM) [41,42] and the learned perceptual image patch similarity (LPIPS) [43] to compare the performance of different methods. These metrics are widely utilized for the evaluation of image super-resolution. Generally, there is a positive correlation between PSNR and SSIM values and the quality of the reconstructed image. Note that LPIPS is more consistent with human visual perception, and a lower LPIPS value indicates a higher perceptual similarity between the reconstructed image and the ground truth.

## 5. Results and Discussions

In this section, we present a thorough experimental evaluation of our proposed method. We benchmark its performance against a diverse set of state-of-the-art super-resolution approaches, encompassing both classic and recent models including ESRGAN [37], BSRGAN [31], Real-ESRGAN [32], DASR [34], A-ESRGAN [44], DAT [45], DRCT [46] and DRSR [47]. The comparison is conducted from two primary perspectives: quantitative analysis using standard metrics and qualitative assessment through visual inspection.

**Figure 7:**
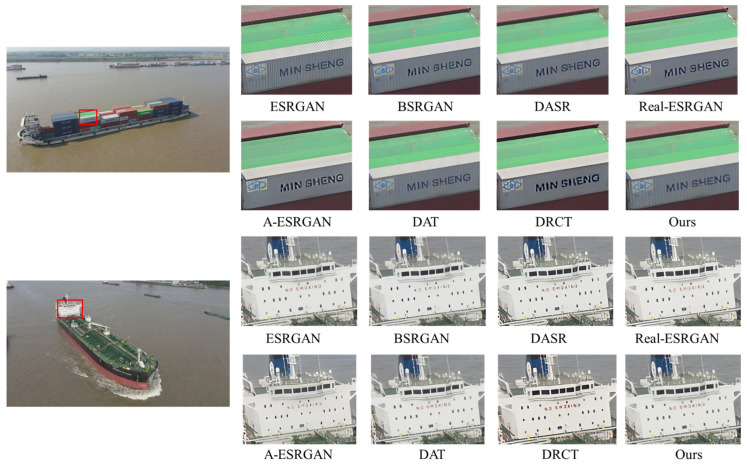
Qualitative comparisons of different methods on images with bicubic degradation. Please zoom in for a better view.

**Figure 8:**
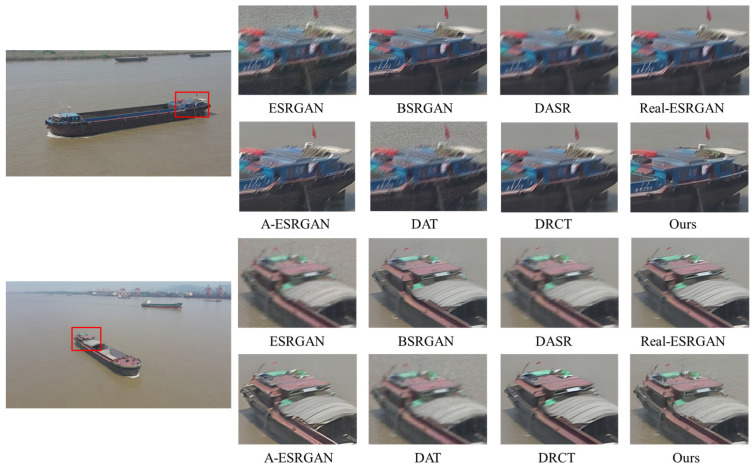
Qualitative comparisons of different methods on images with level 1 degradations. Please zoom in for a better view.

**Figure 9:**
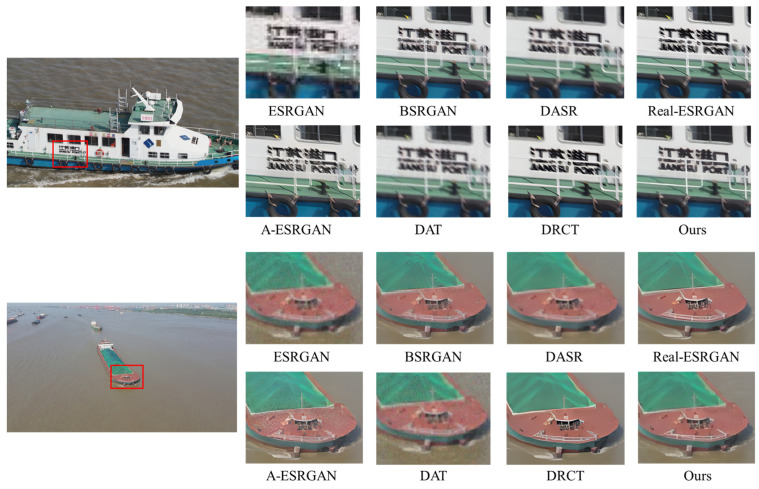
Qualitative comparisons of different methods on images with level 2 degradations. Please zoom in for a better view.

**Figure 10:**
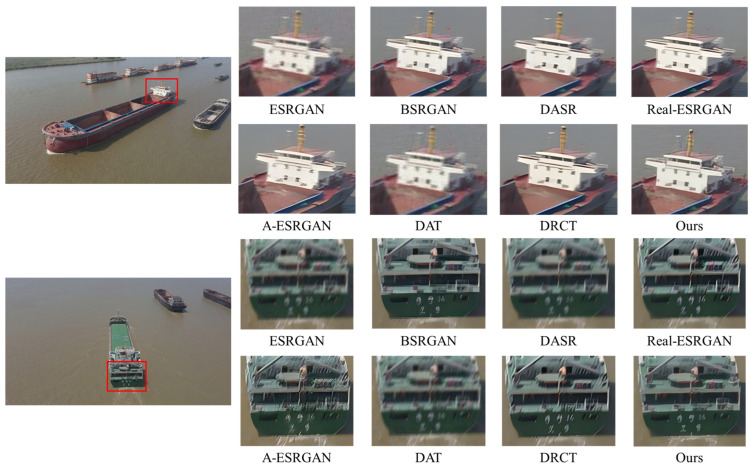
Qualitative comparisons of different methods on images with level 3 degradations. Please zoom in for a better view.

A comprehensive quantitative comparison among these methods is presented in Table 2. For a fair assessment, all methods are evaluated with their respective, officially released pre-trained models. As shown in the table, DAT and ESRGAN achieve great results when evaluated on bicubic-degraded images. However, their performance degrades significantly for other degradation types, as evidenced by a sharp increase in their LPIPS scores. For example, the LPIPS of the DAT method rises from 0.1442 under bicubic degradation to 0.4982 under level 1. This indicates a fundamental limitation of methods trained exclusively on bicubic models. While the blind DRSR super-resolution method outperforms the two methods mentioned above, its LPIPS score remains relatively high in complex degradation scenarios. A-ESRGAN, BSRGAN, Real-ESRGAN, DASR and DRCT outperform the aforementioned methods in terms of LPIPS across the three different degradation scenarios while also exhibiting more robust and stable overall performance, whereas A-ESRGAN exhibits relatively inferior overall performance. However, all these methods still suffer a severe performance drop at degradation level 3, particularly in perceptual quality. In stark contrast, our proposed method demonstrates superior performance, achieving the best overall LPIPS score and ranking among the top performers on the PSNR and SSIM metrics. Taking the level 2 degradation scenario as an example, our method outperforms DASR, with a PSNR gain of 1.13 dB, an SSIM gain of 0.0107, and an LPIPS reduction of 0.0978. Crucially, its performance does not suffer the severe drop observed in other methods, proving its superior stability. These quantitative findings are strongly corroborated by the qualitative comparisons in Figure 7, Figure 8, Figure 9 and Figure 10. As illustrated in Figure 8, Figure 9 and Figure 10, ESRGAN and DAT struggle to effectively suppress noise and restore sharp edges in complex scenes. Their performance is also largely limited to scenarios involving fixed bicubic degradation, as shown in Figure 6. Conversely, BSRGAN, Real-ESRGAN, DASR, A-ESRGAN, and DRCT exhibit a distinct trade-off, as while generating sharp details, they introduce unnatural textures and visual artifacts or result in excessively smooth regions. This compromises the realism of the reconstructed images, as evidenced in Figure 8. In contrast, our method, which employs a “static + dynamic” reconstruction strategy, provides a more balanced and effective solution, yielding images with superior visual clarity and fewer artifacts.

From the results in Table 2, it is evident that DASR represents the state of the art among the baseline methods, second only to our own. Given this and since our method for degradation modeling builds upon and significantly refines the approach used in this model, we regard it as the most relevant and critical baseline for comparison. To ensure a fair and comprehensive evaluation, we adapted DASR by re-training it on our custom degradation datasets. As the results in Table 3 reveal, this adaptation yielded notable improvements for DASR—in particular, a substantial gain in the LPIPS metric. Despite this enhancement, our proposed method still maintains a decisive lead, delivering superior results across the board. We attribute DASR’s initial success to its multi-expert joint optimization strategy. However, our work makes a fundamental advance by integrating static and dynamic reconstruction within a unified framework, which fosters greater robustness and ultimately achieves a higher level of performance.

**Figure 11:**
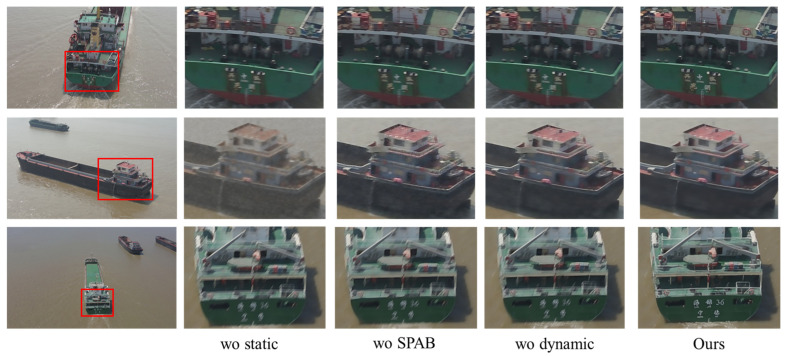
Qualitative results of the ablation study. Please zoom in for a better view.

To further assess the efficacy of the proposed method, we conducted an ablation study using the dataset. The quantitative and qualitative results of the ablation study on images with level 2 degradation are shown in Table 4 and Figure 11, respectively. For the static and dynamic stages, which are executed sequentially, we replaced the removed components with a simple convolutional module to maintain the model’s structural integrity during the experiment. The experimental results clearly demonstrate that the model’s performance degrades most significantly when the static stage is removed. This is because the feature maps generated in this stage serve as the foundational input for the following reconstruction process. Furthermore, the removal of the SPAB attention module compromises the model’s ability to extract fine features, which, in turn, degrades its performance. Likewise, the absence of the dynamic stage impairs the model’s capacity to manage complex types of degradation, leading to a similar performance drop. These findings underscore the distinct and critical role each component plays in the model and demonstrate the effectiveness of our proposed model.

**Figure 12:**
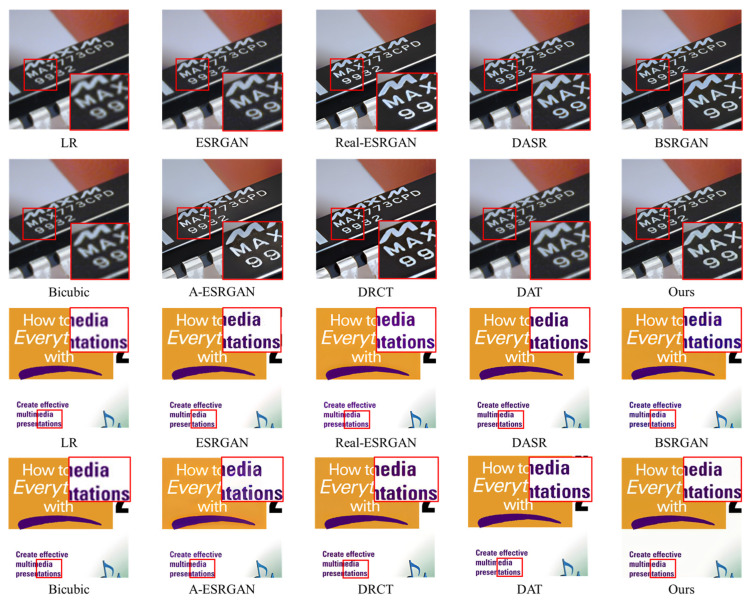
Qualitative comparisons of different methods on real-world images. Please zoom in for a better view.

Furthermore, we conducted experiments on public real-world LR natural images without down-sampling. In this part, experiments were conducted on the images from the public RealSRSet [31]. Figure 12 presents the qualitative results of the performance of different methods on these images with a scale of 4. It can be intuitively seen from the comparison in the figure that our model produces results with visibly clearer details and fewer blurring artifacts, which demonstrates its generalization capacity.

## 6. Conclusions

The expanding application of drones across critical domains is fundamentally constrained by the complex degradations inherent in aerial imagery, which obscure the underlying data structures essential for reliable analysis. For example, in the context of vessel monitoring, image fidelity is a decisive factor for successful pattern recognition, yet degradation can lead to relatively high information entropy. In this study, a novel adaptive super-resolution method for drone-captured images is proposed, which can be applied to the super-resolution reconstruction tasks of drone images in complex scenes. First, to better align with real-world scenes, we optimize the degradation model by integrating the actual flight state of the drones. Second, we introduce a novel network architecture for drone image super-resolution that leverages a “static + dynamic” design to achieve adaptive reconstruction of input images. Finally, we construct a novel dataset of ship images captured by drones and conduct a thorough series of experiments for verification. The results from quantitative and qualitative analyses show that our method produces reconstructed images of higher quality and demonstrates superiority over most current representative algorithms. Furthermore, the restored structural details are expected to significantly enhance the performance of downstream tasks like clustering and object recognition.

Drones are finding increasingly widespread applications, and integrating super-resolution technology to enhance the quality of drone-captured imagery is crucial for improving their performance in downstream tasks that demand high precision. The proposed method is able to address the challenges of SR in complex scenes, featuring an adaptive ability to process diverse input drone images. While our method has shown promising results, its performance is still contingent upon the availability of substantial training data that is well-aligned with the target scenarios. Furthermore, the depth of its exploration of underlying data structures could be enhanced, and its capacity for reconstructing more detailed semantic information for downstream applications deserves further investigation. Looking ahead, a promising direction for future work is to extend super-resolution technology to zero-shot or few-shot learning scenarios, which would further enhance its practical applicability and robustness in real-world conditions. Moreover, the leveraging of large models offers a significant avenue for progress, as their superior generalization allows for the development of super-resolution methods with minimal reliance on large-scale, domain-specific training data.

## Figures and Tables

**Table 1:** Detailed parameter settings of the degradation. [‘a’, ‘b’, ‘b’] denote the resize modes of [area, bilinear, bicubic]; [‘G’, ‘P’] denote the noise types of [Gaussian, Poisson]; R-J and J-R denote the order of resizing and JPEG com-pression, respectively.

Operation	Parameter	Level 1	Level 2	Level 3	
				Stage 1	Stage 2
Blur	kernel size	[7, 21]	[7, 21]	[7, 21]	[7, 21]
	standard deviation	[0.8, 2]	[1.5, 3.5]	[0.5, 3]	[0.5, 2]
	rotation degree	[−π, π]	[−π, π]	[−π, π]	[−π, π]
	sinc kernel size	-	-	-	[7, 21]
	$\omega_{c}$ of sinc kernel	-	-	-	[π/3, π]
Resize	[down, keep]	[0.3, 0.7]	[0.6, 0.4]	[0.3, 0.7]	[0.6, 0.4]
	scale factor	[0.1,0.5]	[0.1, 0.5]	[0.2, 0.5]	[0.2, 0.5]
	resize mode	[‘a’, ‘b’, ‘b’]	[‘a’, ‘b’, ‘b’]	[‘a’, ‘b’, ‘b’]	[‘a’, ‘b’, ‘b’]
Noise	type	[‘G’, ‘P’]	[‘G’, ‘P’]	[‘G’, ‘P’]	[‘G’, ‘P’]
	sigma of Gaussian	[1, 10]	[1, 20]	[1, 20]	[1, 20]
	scale of Poisson	[0.05, 0.5]	[0.05, 1.5]	[0.05, 3]	[0.05, 2]
	gray probability	0.4	0.4	0.4	0.4
JPEG	quality factor	[90, 95]	[50, 95]	[50, 95]	[50, 95]
	operating order	-	-	R-J or J-R	R-J or J-R
	mode of final resize	-	-	[‘a’, ‘b’, ‘b’]	[‘a’, ‘b’, ‘b’]

**Table 2:** Quantitative comparisons of different methods on datasets with different degradations. The PSNR results are calculated in the Y channel of YCbCr space.

Method	Metric	ESRGAN	A-ESRGAN	DAT	DRSR	BSRGAN	Real-ESRGAN	DRCT	DASR	Ours
Bicubic	PSNR	37.57	30.01	40.79	38.65	34.43	33.71	33.05	35.62	38.49
	SSIM	0.9371	0.8648	0.9682	0.9617	0.9242	0.9269	0.9141	0.9479	0.9537
	LPIPS	0.0793	0.2645	0.1442	0.1730	0.1941	0.1689	0.1860	0.1045	0.0744
Level 1	PSNR	28.46	29.92	31.73	31.43	32.42	32.02	31.69	33.19	34.32
	SSIM	0.6244	0.8717	0.8047	0.7883	0.8919	0.8996	0.8970	0.9020	0.9127
	LPIPS	0.5628	0.2930	0.4982	0.4924	0.2536	0.2386	0.2460	0.2732	0.1754
Level 2	PSNR	29.58	29.26	30.98	31.01	31.82	31.31	30.82	32.31	32.85
	SSIM	0.7268	0.8853	0.8267	0.8259	0.8847	0.8890	0.8852	0.8941	0.8985
	LPIPS	0.5027	0.3156	0.4908	0.4812	0.2671	0.2590	0.2711	0.3020	0.1933
Level 3	PSNR	30.61	28.50	30.75	30.65	30.18	29.64	29.69	30.85	31.28
	SSIM	0.8674	0.8440	0.8746	0.8665	0.8606	0.8579	0.8690	0.8801	0.8799
	LPIPS	0.4660	0.3427	0.5135	0.4827	0.3172	0.3113	0.3068	0.3389	0.2317

**Table 3:** Quantitative comparisons of re-trained DASR and our method.

Method	Metric	Bicubic	Level 1	Level 2	Level 3
DASR	PSNR	35.62	33.19	32.31	30.85
	SSIM	0.9479	0.9020	0.8941	0.8801
	LPIPS	0.1045	0.2732	0.3020	0.3389
DASR-re	PSNR	38.30	33.61	32.17	31.05
	SSIM	0.9511	0.9089	0.8943	0.8827
	LPIPS	0.0817	0.2229	0.2506	0.2942
Ours	PSNR	38.49	34.32	32.85	31.28
	SSIM	0.9537	0.9127	0.8985	0.8799
	LPIPS	0.0744	0.1754	0.1933	0.2317

**Table 4:** Quantitative results of the ablation study.

Model	PSNR	SSIM	LPIPS
wo static	31.53	0.8881	0.2455
wo SPAB	32.08	0.8894	0.2257
wo dynamic	32.20	0.8914	0.2138
Ours	32.85	0.8985	0.1933

## Data Availability

Due to privacy regulations and continued use for a follow-up project, part of the data presented in this study are available upon request from the corresponding author.
